# Opioid Modulation of Major Neurotransmitter Systems: Acute, Chronic, and Withdrawal Effects

**DOI:** 10.1111/ejn.70735

**Published:** 2026-10-30

**Authors:** Daniela Novakova, Eliska Pritulova, Jiri Novotny

**Affiliations:** ^1^ Department of Physiology, Faculty of Science Charles University Prague Czechia; ^2^ First Faculty of Medicine Charles University Prague Czechia

**Keywords:** acetylcholine, dopamine, GABA, glutamate, neurotransmitters, opioid dependence, opioid withdrawal, opioids, serotonin

## Abstract

Opioids are widely used in clinical practice for the management of moderate‐to‐severe acute and chronic pain. However, their use is associated with the development of tolerance and physical dependence, often followed by a withdrawal syndrome upon discontinuation. The mechanisms underlying opioid dependence and withdrawal are highly complex and remain incompletely understood. These processes involve multiple regions of the central nervous system and are accompanied by widespread alterations in neuronal signaling pathways. Neurotransmitters, which are essential for communication between neurons, play a critical role in opioid addiction and withdrawal. In this review, we provide a comprehensive overview of the major neurotransmitter systems, their involvement, and the changes that occur during acute and chronic opioid exposure. Particular attention is also given to the consequences of opioid discontinuation. Overall, this review summarizes current knowledge of the molecular mechanisms underlying both the acute and long‐term effects of opioids on key neurotransmitter systems in the brain.

## Graphical abstract

Opioids are widely used but also frequently misused drugs that affect multiple neurotransmitter systems, including dopaminergic, glutamatergic, GABAergic, serotonergic, and cholinergic pathways. Opioid‐induced neuroadaptations depend on the duration of drug exposure, with distinct changes also emerging following drug discontinuation. Dysregulation of these neurotransmitter systems contributes to the development and maintenance of opioid use disorder. This review summarizes current knowledge of the effects of acute and chronic opioid exposure, as well as withdrawal, on major neurotransmitter systems in the brain.

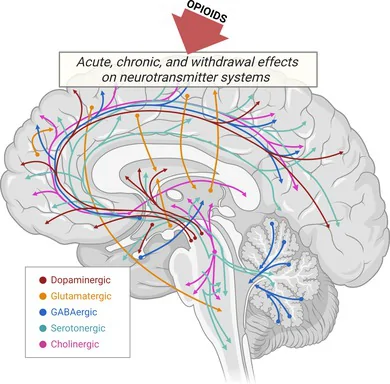

**Abbreviations**
AC: adenylyl cyclase
AcCoA: acetyl‐coenzyme A
ACh: acetylcholine
AChE: acetylcholinesterase
AMPAR: α‐amino‐3‐hydroxy‐5‐methyl‐4‐isoxazolepropionic acid receptor
AMY: amygdala
CB: cerebellum
Ch: choline
ChT: choline transporter
ChAT: choline acetyltransferase
CNS: central nervous system
COMT: catechol‐O‐methyltransferase
CPu: caudate putamen
Ctx: cerebral cortex
DA: dopamine
DAG: diacylglycerol
DAT: dopamine transporter
DβH: dopamine β‐hydroxylase
DOPAC: 3,4‐dihydroxyphenylacetic acid
DOR: δ‐opioid receptor
DRN: dorsal raphe nucleus
EAAT: excitatory amino acid transporter
GABA: γ‐aminobutyric acid
GAD: glutamate decarboxylase
GAT: GABA transporter
GIRK: G protein–activated inwardly rectifying potassium channel
GDH: glutamate dehydrogenase
GLN: glutamine
GLS: glutaminase
GLT: glutamate transporter
GLU: glutamate
GPCR: G protein–coupled receptor
GS: glutamine synthetase
HIP: hippocampus
HVA: homovanillic acid
iGluRs: ionotropic glutamate receptors
IP₃: inositol 1,4,5‐trisphosphate
KOR: κ‐opioid receptor
LC: locus coeruleus
L‐DOPA: L‐3,4‐dihydroxyphenylalanine
LDTg: laterodorsal tegmental nucleus
MAO: monoamine oxidase
MAPK: mitogen‐activated protein kinase
mGluRs: metabotropic glutamate receptors
MOR: μ‐opioid receptor
NAc: nucleus accumbens
NMDAR: N‐methyl‐D‐aspartate receptor
NE: norepinephrine
OR: opioid receptor
OUD: opioid use disorder
PAG: periaqueductal gray
PFC: prefrontal cortex
PKA: protein kinase A
PKC: protein kinase C
PLC: phospholipase C
SERT: serotonin transporter
TCA: tricarboxylic acid
TPH: tryptophan hydroxylase
VGAT: vesicular GABA transporter
VTA: ventral tegmental area
5‐HIAA: 5‐hydroxyindoleacetic acid
5‐HT: 5‐hydroxytryptamine (serotonin)
αKG: α‐ketoglutarate

## 1 Introduction

Communication between individual cells is a fundamental feature of multicellular organisms. In the nervous system, this function is primarily mediated by neurotransmission. Neurotransmitters are endogenous signaling molecules that transmit information from presynaptic neurons to target postsynaptic cells (Rizo 2018). These molecules are typically small and include acetylcholine (ACh), amino acids such as glutamate—the principal excitatory neurotransmitter—and their derivatives, such as γ‐aminobutyric acid (GABA), the primary inhibitory neurotransmitter. In addition, neurotransmitters comprise monoaminergic mediators, which are classified into catecholamines (dopamine [DA], norepinephrine [NE], and epinephrine), synthesized from tyrosine and characterized by a catechol moiety, and indolamines (tryptamines), synthesized from tryptophan and containing an indole structure, with serotonin as a key representative (Bowery and Smart 2006; Kawai et al. 2007; Ko and Strafella 2012; Rapport et al. 1948). Interestingly, most neurotransmitters, including ACh, DA, NE, serotonin, glutamate, and GABA, can also function as neuromodulators. Unlike classical neurotransmitters, which typically produce rapid excitatory or inhibitory effects in target cells and thereby enable fast communication within neural circuits, neuromodulators primarily influence the excitability of neurons, the strength of synaptic transmission, and the way neural networks respond to incoming signals. Their effects are often more prolonged and can extend beyond the immediate synaptic environment. Whether a signaling molecule functions primarily as a neurotransmitter or a neuromodulator depends on several factors, including its site of release, the receptors it activates, the spatial and temporal dynamics of signaling and the extent of its effects within the surrounding neural network. Thus, the same signaling molecule may function as either a neurotransmitter or a neuromodulator depending on the cellular and physiological context (Özcete et al. 2024). It is worth noting in this context that endogenous opioids are neuropeptides that function primarily as neuromodulators, whereas opioid drugs exert similar neuromodulatory effects by mimicking the actions of endogenous opioids. By modulating neuronal activity and synaptic transmission, opioids can substantially influence brain function (Reeves et al. 2022).

Opioids are best known for their potent analgesic effects, acting via opioid receptors (ORs) in the central nervous system (CNS) to suppress pain transmission. Although opium has been known since ancient Sumerian times, the significance of opioids in clinical pharmacology increased following the isolation of morphine from the opium poppy in 1806 (Brook et al. 2017). Today, opioids remain an integral component of acute and chronic pain management. They can be administered via multiple routes, including epidural and intrathecal delivery, and are used not only in anesthesia but also in the treatment of conditions such as diarrhea and cough. Despite their therapeutic benefits, opioids are widely misused due to their psychotropic effects (Volkow et al. 2018). One of the most serious consequences of opioid use is addiction, characterized by compulsive drug‐seeking behavior and withdrawal symptoms upon cessation, including insomnia, anxiety, and depression (Ghasemi et al. 2015; Kosten and Baxter 2019; Listos et al. 2013). The development of tolerance often necessitates escalating doses to achieve the same therapeutic effect (Al‐Hasani and Bruchas 2011). Moreover, the risk of respiratory depression—a potentially fatal adverse effect—varies among opioids due to differences in their pharmacokinetic and pharmacodynamic properties. Highly potent opioids pose the greatest risk for overdose and dependence (Burgraff et al. 2023). Importantly, prolonged opioid exposure induces complex, brain region–specific molecular alterations that are highly variable and may persist long after drug discontinuation (Drastichova et al. 2021; Hejnova et al. 2024; Ujcikova et al. 2021). Although the effects of opioids on neurotransmission have been extensively studied, their impacts on individual neurotransmitter systems have not yet been systematically integrated. A comprehensive understanding of these interactions is essential for developing more effective therapeutic strategies for opioid use disorder (OUD), particularly for addressing opioid addiction and withdrawal symptoms.

## 2 Opioid Pharmacology and Mechanisms of Action

### 2.1 Opioids and the Opioid System

Opioid substances can be classified into endogenous opioids—such as endorphins, enkephalins, dynorphins, and endomorphins—and exogenous opioids. The endogenous opioid system plays a critical role in regulating reward processing, mood, motivation, learning and memory, gastrointestinal function, immune responses, and analgesia (Higginbotham et al. 2022). Endogenous opioids are present in both the central and peripheral nervous systems. Exogenous opioids, in contrast, are administered externally and bind to the same receptors as endogenous opioids. These compounds are further categorized into naturally occurring opioid alkaloids (e.g., morphine, codeine, and thebaine) and semisynthetic or synthetic opioids (e.g., heroin, oxycodone, and fentanyl) (Drewes et al. 2013). Opioids exert their effects primarily through three classical OR subtypes: mu (μ, MOR), delta (δ, DOR), and kappa (κ, KOR). Each receptor subtype contributes uniquely to the modulation of analgesia, reward, and addictive behaviors. Activation of these receptors alters neuronal excitability and neurotransmission, thereby influencing a wide array of physiological and psychological functions (Reeves et al. 2022; Stevens 2009).

### 2.2 ORs and Mechanism of Action

The concept of ORs was first proposed in the 1950s (Beckett and Casy 1954), with stereospecific binding sites subsequently identified in the brain during the 1970s. These studies demonstrated that both exogenous ligands and naturally occurring opioid‐like peptides exert their effects by binding to distinct ORs (Snyder and Childers 1979). The classical ORs—MOR, DOR, and KOR—are members of the G protein–coupled receptor (GPCR) family. A fourth receptor, the nociceptin/orphanin FQ receptor, is considered a nonclassical member of the OR family (Dhawan et al. 1996). Agonist binding to these receptors triggers intracellular signaling cascades that induce cellular hyperpolarization, ultimately reducing neuronal excitability and modulating pain perception (Cox et al. 2015; Pathan and Williams 2012).

Structurally, ORs contain seven transmembrane domains, three extracellular and three intracellular loops, an extracellular N‐terminal domain, and an intracellular C‐terminal domain. While these receptors share a high degree of homology, each subtype exhibits a distinct pharmacological profile (Raynor et al. 1994). ORs are widely expressed throughout the central and peripheral nervous systems. Initial localization studies in 1973 revealed their presence in brain regions involved in pain transmission, including the thalamus, rostroventral medulla, and periaqueductal gray (PAG), as well as in key components of the reward circuitry, such as the nucleus accumbens (NAc) and ventral tegmental area (VTA) (Pert and Snyder 1973). ORs are also found in additional brain regions, including the hypothalamus, amygdala (AMY), ventral pallidum, globus pallidus, raphe nuclei, and hippocampus (HIP) (George et al. 1994; Kitchen et al. 1997; Mansour et al. 1995). The distribution of OR subtypes varies across brain regions. MOR is highly expressed throughout the forebrain, midbrain, and hindbrain, with dense expression in the cerebral cortex (Ctx), caudate putamen (CPu), NAc, thalamus, HIP, AMY, colliculi, nucleus tractus solitarius, spinal trigeminal nucleus, and dorsal horn (Mansour et al. 1987). KOR is concentrated in the CPu, NAc, AMY, hypothalamus, and nucleus tractus solitarius (Clarke et al. 2001; Jomary et al. 1988), whereas DOR is less abundant in the CNS, with prominent expression in the olfactory bulb, Ctx, CPu, NAc, and AMY (Goody et al. 2002). In peripheral tissues, ORs are present in the gastrointestinal and respiratory systems (Wittert et al. 1996).

Binding of an opioid ligand induces conformational changes in the associated heterotrimeric G protein, composed of α, β, and γ subunits. Upon activation, the Gα subunit exchanges GDP for GTP and dissociates from the Gβγ dimer. Both components contribute to intracellular signaling pathways (Childers et al. 1979; Svoboda et al. 2004). While stimulatory G proteins (Gs) activate adenylyl cyclase (AC) and increase intracellular cAMP levels, inhibitory G proteins (Gi) suppress AC activity, resulting in reduced cAMP production. This decrease leads to diminished activation of protein kinase A (PKA) and reduced phosphorylation of the cAMP response element‐binding protein (CREB) (Fleming et al. 1992).

OR activation engages two principal signaling pathways: the G protein–dependent pathway and the β‐arrestin pathway. The β‐arrestin pathway regulates receptor desensitization and internalization (Bohn et al. 2000; Whistler et al. 1999; Zhang et al. 1998), whereas the G protein pathway mediates classical intracellular signaling events that vary depending on receptor subtype (Ho et al. 2018; Mafi et al. 2021). These pathways influence multiple downstream targets, including AC, phosphatidylinositol 3‐kinase, MAPK cascades, calcium channels, and G protein–activated inwardly rectifying potassium (GIRK) channels (Al‐Hasani and Bruchas 2011). OR predominantly couples to Gi/o proteins, thereby exerting inhibitory effects. Activation of Gi inhibits AC, while the Gβγ subunits can directly interact with GIRK channels, promoting potassium efflux and neuronal hyperpolarization (Montandon et al. 2016). Additionally, the Gβγ complex can inhibit voltage‐gated calcium channels, reducing calcium influx into the cell (Xie et al. 2007).

## 3 Overview of Major Neurotransmitter Systems

### 3.1 The Dopaminergic System

Dopamine (DA) is one of the brain's key neurotransmitters, regulating behavior, reward, learning, and memory processes (Ranjbar‐Slamloo and Z. Fazlali 2020). It belongs to the catecholamine group, a class of biogenic amines characterized by a 3,4‐dihydroxyphenyl (catechol) structure. Its biosynthesis originates from L‐tyrosine, which is first hydroxylated to L‐3,4‐dihydroxyphenylalanine (L‐DOPA) and subsequently decarboxylated to dopamine by aromatic L‐amino acid decarboxylase. DA can be further converted into norepinephrine (NE) via dopamine‐β‐hydroxylase (DβH), and NE may be methylated by phenylethanolamine N‐methyltransferase to form epinephrine. Dopamine is metabolized by monoamine oxidase (MAO) to 3,4‐dihydroxyphenylacetic acid (DOPAC), which is subsequently converted by catechol‐O‐methyltransferase (COMT) into homovanillic acid (HVA) (Nagatsu 2007).

Within the CNS, DA is synthesized by dopaminergic neurons that are widely distributed throughout the brain and project to multiple target regions, thereby regulating a broad spectrum of physiological functions. Dopaminergic neurons are primarily located in the substantia nigra, projecting to the CPu and forming the nigrostriatal pathway, which is essential for motor control. Additional dopaminergic populations reside in the VTA, projecting to the NAc, AMY, and prefrontal cortex (PFC). This mesolimbic pathway plays a key role in emotional regulation, motivation, reward processing, and memory formation. Dopaminergic neurons are also present in the hypothalamus, where they project to the pituitary gland and modulate endocrine function (Gerfen 1992; Grimm et al. 2004; Kobayashi 2001; Le Moal and Simon 1991).

DA exerts its effects via dopamine receptors, which belong to the GPCR family. These receptors are classified into five subtypes (D1–D5), each encoded by distinct genes and exhibiting specific functional roles and regional distributions (Kebabian and Calne 1979). Based on G protein coupling, dopamine receptors are divided into two major groups. D1‐like receptors (D1 and D5) couple to stimulatory Gs proteins, leading to activation of AC and increased cAMP levels. This activates PKA, which regulates transcription factors and other intracellular targets. In addition, these receptors can activate phospholipase C (PLC), resulting in the production of inositol 1,4,5‐trisphosphate (IP₃) and diacylglycerol (DAG), thereby promoting intracellular calcium release and activation of protein kinase C (PKC). These receptors are widely distributed in the striatum, NAc, olfactory bulb, and substantia nigra, where they contribute to reward processing, motor control, learning, and memory (Bueschbell et al. 2021; Jaber et al. 1996). In contrast, D2‐like receptors (D2, D3, and D4) couple to inhibitory Gi proteins, resulting in suppression of AC activity and modulation of ion channels, including activation of potassium channels. These receptors are involved in cognitive processes, attention, sleep regulation, and neuronal development and are primarily expressed in the striatum, globus pallidus, NAc, HIP, AMY, and Ctx (Jaber et al. 1996; Lacey et al. 1987; Salvi et al. 2016).

### 3.2 The Glutamatergic System

Glutamate is a nonessential amino acid that plays a central role in amino group transfer and serves as the principal excitatory neurotransmitter in the CNS. It can be synthesized via reversible catalysis by glutamate dehydrogenase (GDH) from α‐ketoglutarate, an intermediate of the Krebs cycle, or through enzymatic conversion from glutamine (Mathioudakis et al. 2019; Reitzer 2004). Following its release into the synaptic cleft, glutamate is taken up by glial cells and converted into glutamine by glutamine synthetase (GS). Glutamine is subsequently transported back to neurons, where it is reconverted into glutamate, thereby completing the glutamate–glutamine cycle (Palmada and Centelles 1998). Alternatively, glutamate may be cleared via excitatory amino acid transporters or bind to its receptors to exert its effects.

Glutamate acts on two major classes of receptors: ionotropic receptors (iGluRs), which function as ligand‐gated ion channels, and metabotropic receptors (mGluRs), which are GPCRs. Both receptor classes are critically involved in numerous physiological and pathophysiological processes within the CNS. iGluRs are tetrameric complexes composed of four subunits forming an ion‐conducting pore. They are classified into four main groups: AMPA receptors (GluA1–GluA4), kainate receptors (GluK1–GluK5), NMDA receptors (GluN1, GluN2A–GluN2D, GluN3A, GluN3B), and δ receptors (GluD1 and GluD2) (Traynelis et al. 2010). These receptors mediate fast excitatory neurotransmission and are essential for processes such as synaptic plasticity, learning, and memory. Their expression is developmentally regulated and region‐specific. AMPA receptors are widely expressed in the Ctx, HIP, and cerebellum (CB) and are often colocalized with NMDA receptors. In contrast, kainate receptors are predominantly expressed on inhibitory interneurons, particularly within hippocampal circuits (Fisahn et al. 2004; Traynelis et al. 2010). mGluRs are subdivided into three groups based on their signaling mechanisms and sequence homology. Group I mGluRs are predominantly neuronal, and their activation stimulates PLC and AC, as well as the MAPK signaling pathways. Group II mGluRs are expressed in both neurons and astrocytes (notably mGluR3) and inhibit AC and calcium channels while activating potassium channels. Group III mGluRs are widely distributed in neurons (e.g., mGluR4 and mGluR7) and in the retina (mGluR6); their activation is associated with inhibition of AC and calcium channels, activation of potassium channels, and modulation of cGMP signaling (Niswender and Conn 2010). The broad distribution of mGluRs underscores their involvement in neural development, synaptic plasticity, and a range of neurological and psychiatric disorders, including Alzheimer's disease, Parkinson's disease, schizophrenia, addiction, anxiety, and depression (Niswender and Conn 2010; Sherman 2014).

### 3.3 The GABAergic System

Gamma‐aminobutyric acid (GABA) is the principal inhibitory neurotransmitter in the adult CNS (Vargas 2018). It is synthesized from glutamate by glutamate decarboxylase (GAD). Following synthesis, GABA is stored in synaptic vesicles and released into the synaptic cleft in response to neuronal stimulation. After release, GABA is taken up by GABA transporters (GATs) and metabolized to succinate via the sequential actions of GABA transaminase and succinate semialdehyde dehydrogenase or it binds to its receptors to exert inhibitory effects (Schousboe et al. 2004).

Three main classes of GABA receptors have been identified: GABA_A_, GABA_A_‐ρ, and GABA_B_ receptors. GABA_A_ and GABA_A_‐ρ receptors are ligand‐gated chloride ion channels, whereas GABA_B_ receptors are GPCRs that inhibit AC activity and reduce calcium influx, thereby decreasing the release of excitatory neurotransmitters (Bormann 2000; Naffaa et al. 2017; Sigel and Steinmann 2012). GABA receptors are widely distributed throughout the CNS and play essential roles in numerous physiological processes, including sleep regulation, neurodevelopment, and neuroendocrine function (Chebib et al. 2004). In addition, GABAergic signaling influences peripheral systems such as the cardiovascular and reproductive systems (Kah et al. 1992; Vargas 2018; Wang et al. 2008). GABAergic neurons located in the rostromedial tegmental nucleus exert inhibitory control over dopaminergic neurons in the VTA and are involved in nociceptive processing as well as opioid‐mediated analgesia (Taylor et al. 2019). GABAergic neurons contribute to the formation and modulation of neural circuits linking regions such as the Ctx, HIP, limbic system, and pituitary gland. Dysregulation of the GABAergic system has been implicated in various pathological conditions, including anxiety and stress‐related disorders, pain, addiction, and withdrawal syndromes (Smith and Rudolph 2012; Stachowicz et al. 2008).

### 3.4 The Serotonergic System

The principal neurotransmitter of the serotonergic system is the indoleamine 5‐hydroxytryptamine (5‐HT, serotonin). Its synthesis begins with the essential amino acid L‐tryptophan, which enters the kynurenine metabolic pathway (Höglund et al. 2019). In serotonergic neurons, tryptophan hydroxylase (TPH) catalyzes the conversion of L‐tryptophan to 5‐hydroxy‐L‐tryptophan, which is subsequently decarboxylated to serotonin by aromatic L‐amino acid decarboxylase. Serotonin is primarily metabolized by MAO to 5‐hydroxyindoleacetic acid, a process that may occur directly within the synaptic cleft (Adaway et al. 2016; Höglund et al. 2019).

Serotonergic neurons are primarily located in the raphe nuclei and project extensively to numerous brain regions, including the Ctx, AMY, CB, HIP, thalamus, and hypothalamus. In addition to its central role, serotonin is also synthesized in large quantities in the gastrointestinal tract by enterochromaffin cells. Owing to its widespread distribution, serotonin regulates a diverse range of physiological functions, including mood, cognition, aggression, sleep, thermoregulation, cardiovascular function, and pain perception (Cao et al. 2017; Charnay and Léger 2010).

Serotonin exerts its effects through a diverse family of GPCRs, comprising more than 15 subtypes grouped into seven major classes (5‐HT₁–5‐HT₇) based on structural and pharmacological characteristics (Pithadia and Jain 2009). The 5‐HT₁ receptor family generally inhibits AC and is expressed in regions such as the raphe nuclei, HIP, striatum, and Ctx, as well as in peripheral tissues including smooth muscle and the uterus (Hoyer et al. 2002). The 5‐HT_2_ receptor family activates PLC and is distributed in the Ctx, CB, hypothalamus, and peripheral tissues such as smooth muscle and platelets (Conn and Sanders‐Bush 1984). The 5‐HT₃ receptor is a ligand‐gated ion channel that modulates the release of other neurotransmitters and is primarily localized in the area postrema and visceral afferent neurons (Alhaider et al. 1991). The 5‐HT₄ receptor activates AC and is found in the HIP and gastrointestinal tract. The 5‐HT₅ receptor is expressed in the olfactory bulb and habenula, although its function remains incompletely understood (Pithadia and Jain 2009). The 5‐HT₆ receptor is predominantly expressed in the NAc, olfactory tubercle, striatum, and HIP, where it regulates glutamatergic and cholinergic neurotransmission and contributes to the modulation of mood and anxiety (Riccioni 2010). The 5‐HT₇ receptor is located in thalamocortical and limbic regions, suggesting a role in sensory processing and affective behavior (Hoyer et al. 2002).

### 3.5 The Cholinergic System

ACh is a classical neurotransmitter synthesized in cholinergic neurons from choline and acetyl‐coenzyme A by choline acetyltransferase. It is rapidly degraded in the synaptic cleft by acetylcholinesterase (AChE) (Potter 1970). Cholinergic neurons can be broadly categorized into projection neurons, located in the basal forebrain and brainstem, and interneurons, primarily found in the striatum and NAc. Basal forebrain projection neurons innervate the Ctx, HIP, and AMY, where they play essential roles in cognition, learning, and arousal. Brainstem cholinergic neurons project to midbrain and brainstem structures, including the VTA, where they modulate dopaminergic activity (Ananth et al. 2023).

ACh acts on two major receptor classes: nicotinic receptors, which are ligand‐gated ion channels, and muscarinic receptors, which are GPCRs. Nicotinic receptors are subdivided into muscular (N1) and neuronal (N2) types. In the CNS, they are primarily localized presynaptically in regions such as the Ctx, substantia nigra, NAc, CPu, HIP, AMY, and striatum, where they regulate the release of multiple neurotransmitters, including DA, NE, glutamate, and GABA (Papke 2014). Muscarinic receptors are divided into five subtypes (M1–M5). M1, M3, and M5 receptors are coupled to Gq proteins and activate PLC signaling pathways, whereas M2 and M4 receptors are coupled to Gi proteins and inhibit AC activity. Within the CNS, M1, M4, and M5 are the predominant subtypes. M1 receptors are highly expressed in the Ctx, HIP, and striatum, whereas M4 and M5 receptors are mainly localized in the HIP and substantia nigra (Jiang et al. 2014; Lebois et al. 2018).

The cholinergic system plays a pivotal role in modulating both dopaminergic and glutamatergic neurotransmission, particularly within the VTA, NAc, and PFC. In the VTA, activation of both nicotinic and muscarinic receptors enhances dopaminergic and glutamatergic signaling, thereby influencing reward‐related processes. In the NAc, cholinergic interneurons integrate cortical and subcortical inputs, contributing to reward processing and behavioral reinforcement. In the PFC, cholinergic signaling is critically involved in cognitive aspects of addiction, including decision‐making, attention, and impulse control (Jensen et al. 2018; Mohebi et al. 2023).

## 4 Effects of Opioids on Neurotransmitter Systems

### 4.1 Opioids and the Dopaminergic System

#### 4.1.1 Effects of Acute Administration

Acute opioid administration exerts profound effects on the dopaminergic system through both direct and indirect mechanisms. Opioids can directly stimulate dopaminergic neurons in key regions of the brain's reward circuitry, including the VTA, striatum, PFC, AMY, HIP, and other limbic structures. This activation reinforces reward processing and increases motivation for continued opioid use (Ahmadi Soleimani et al. 2018; Matthews and German 1984). Indirectly, opioids act via activation of MORs located on GABAergic neurons in the VTA. This reduces GABA release, thereby disinhibiting dopaminergic neurons and enhancing dopaminergic transmission, a mechanism strongly associated with addiction development (Doyle and Mazei‐Robison 2021; Johnson and North 1992; Matsui et al. 2014; Ting‐A‐Kee and van der Kooy 2012). Activation of VTA neurons also increases DA release in the HIP, influencing hippocampal plasticity. Notably, NMDA receptor activation within the VTA–HIP pathway and D1 receptor signaling in the HIP are critical for both rewarding and aversive drug effects, highlighting the role of dopaminergic output in drug‐associated learning and memory (Hu et al. 2014).

D1 receptor signaling is closely linked to DARPP‐32, a key integrator of neurotransmission. Acute morphine administration induces DARPP‐32 phosphorylation, inhibiting protein phosphatase‐1 and preventing dephosphorylation of downstream targets such as glutamate receptors and ion channels. This enhances cAMP signaling cascades (Greengard et al. 1999; Hemmings et al. 1984). Although MOR or KOR agonists do not appear to alter D1 receptor numbers, acute administration has been associated with upregulation of D2 receptors, suggesting complex dopaminergic adaptations (Rooney et al. 1991).

Acute morphine increases extracellular DA levels in reward‐related regions, accompanied by elevated tyrosine levels and increased tyrosine hydroxylase activity, enhancing DA synthesis (García‐Pérez et al. 2013; Pothos et al. 1991). Similarly, MOR agonists (e.g., DAMGO) and DOR agonists (e.g., DPDPE) increase DA and its metabolites (DOPAC, HVA), whereas KOR agonists (e.g., E‐2078) decrease DA levels in the NAc. These findings support opposing roles of MOR and KOR signaling within the reward system (Narita et al. 1993; Spanagel et al. 1990; Xi et al. 1998). Their interaction within the mesolimbic pathway may underlie opioid reinforcement (Xi 2002).

Notably, a single dose of the KOR agonist U‐69593 significantly increased DA uptake in the NAc of male Sprague Dawley rats, indicating enhanced dopamine transporter (DAT) function (Thompson et al. 2000). Otherwise, there are no reports demonstrating effects of acute opioid administration on DAT function or metabolic enzymes. Consistent with this, acute methadone administration did not alter MAO activity in different brain regions (Kaufmann et al. 1983; Simantov 1993).

#### 4.1.2 Effects of Chronic Administration

Chronic opioid exposure enhances dopaminergic activity by increasing neuronal firing and inducing long‐term plasticity within the mesolimbic pathway (Simmons et al. 2019). MOR activation on GABAergic neurons continues to play a central role in shaping reward‐related behavior (Galaj et al. 2020; Galaj and Xi 2021). Elevated DA levels are accompanied by increased concentrations of its metabolites, DOPAC and HVA (Pothos et al. 1991; Spampinato et al. 1985).

Although many MOR agonists similarly elevate DA levels, their effects on metabolic enzymes differ. Morphine, oxycodone, and codeine do not affect MAO activity, whereas methadone, tramadol, and dextromethorphan inhibit MAO, prolonging dopaminergic signaling (Gillman 2005; Mercuri et al. 1997; VanderWende and Spoerlein 1979). These effects are partly mediated through nonopioid mechanisms. In addition to MAO, opioids also influence other enzymes involved in catecholamine metabolism. Genetic variability in COMT has been associated with differential responses to opioids, with reduced COMT activity leading to increased catecholamine levels and heightened pain sensitivity (Duan et al. 2020; Nielsen et al. 2017). DβH activity has also been shown to increase following chronic morphine exposure, further altering catecholamine metabolism (Anderson and Slotkin 1975).

Chronic opioid use additionally induces alterations in receptor expression and intracellular signaling pathways. D1 receptor activity contributes to ΔFosB induction, while reduced mGluR2 sensitivity and altered D2/D3 receptor expression modulate mesolimbic signaling in a maladaptive direction (Maldonado et al. 1997; Muller and Unterwald 2005; Narita et al. 2003; Nestler 2008). D4 receptor signaling has also been implicated in opioid‐induced neuroplasticity (Lüscher et al. 1997; Rivera et al. 2021; Samani et al. 2023). In parallel, chronic opioid exposure impairs dopamine DAT function, with reduced transporter activity observed in both animal models and human opioid users (Liu et al. 2013; Shi et al. 2008; Simantov 1993; Thompson et al. 2000).

#### 4.1.3 Effects of Opioid Withdrawal

Opioid withdrawal is characterized by decreased dopamine and metabolite levels in key reward regions, such as the VTA and NAc, contributing to negative affective states (Acquas et al. 1991; Rada et al. 2004; Rossetti et al. 1992). In addition, experimental studies have shown that morphine withdrawal markedly alters spine density in the NAc, further impairing already reduced dopaminergic transmission (Diana et al. 2006). During withdrawal, a compensatory dopaminergic hypersensitivity emerges, driven primarily by altered D2 receptor responsiveness rather than receptor upregulation (Gianutsos et al. 1974; Strickland et al. 2022).

Recently, epigenetic mechanisms, including histone H3 dopaminylation, have been implicated in long‐lasting transcriptional adaptations and increased vulnerability to relapse (Fulton et al. 2022; Cheng et al. 2023; Stewart et al. 2023). H3 dopaminylation at glutamine 5 (Q5), induced by prolonged heroin exposure, has been identified as an epigenetic mechanism in the VTA that recruits specific transcriptional programs, thereby altering dopaminergic neurotransmission and contributing to drug‐seeking behavior during prolonged abstinence (Fulton et al. 2022; Cheng et al. 2023; Stewart et al. 2023).

The effects of acute and chronic opioid administration, as well as withdrawal, on the dopaminergic system are summarized in Table 1 and illustrated in Figure 1.

**TABLE 1:** Effects of opioids on the dopaminergic system.

Category	Acute administration	Chronic administration	Withdrawal	Reference
Neurotransmitter levels	↑ DA, DOPAC, and HVA in the mesolimbic areas (MOR and DOR agonists) ↓ DA levels in the NAc (KOR agonists)	↑ DA, DOPAC, and HVA in the mesolimbic areas (MOR agonists)	↓ DA, DOPAC, HVA in the VTA and NAc (MOR agonists)	Spampinato et al. (1985), Spanagel et al. (1990), Acquas et al. (1991), Pothos et al. (1991), Rossetti et al. (1992), Narita et al. (1993), Xi et al. (2004), Simmons et al. (2019)
Receptor expression and function	↑ DR signaling → enhanced cAMP cascade ↑ D2R signaling	↑ D2, D3, D4 R expression (MOR agonists)	↑ Sensitivity of D2R (MOR agonists)	Hemmings et al. (1984), Lüscher et al. (1997), Maldonado et al. (1997), Rooney et al. (1991), Greengard et al. (1999), Narita et al. (2003), Spangler et al. (2003), Rivera et al. (2021)
Transporters	↑ DAT function in the NAc (KOR agonists)	↓ DAT function in the VTA and NAc (MOR agonists)		Anderson and Slotkin (1975), Simantov (1993), Shi et al. (2008), Thompson et al. (2000), Liu et al. (2013)
Enzymes	↑ TH activity and ↑ DA synthesis (MOR agonists)	↓ MAO activity (MOR agonists, e.g., methadone tramadol) ↑ DBH activity (MOR agonists)		Gauchy et al. (1973), Anderson and Slotkin (1975), VanderWende and Spoerlein (1979), Pothos et al. (1991), Mercuri et al. (1997), Gillman (2005), Pathan and Williams (2012), García‐Pérez et al. (2013)

**FIGURE 1:**
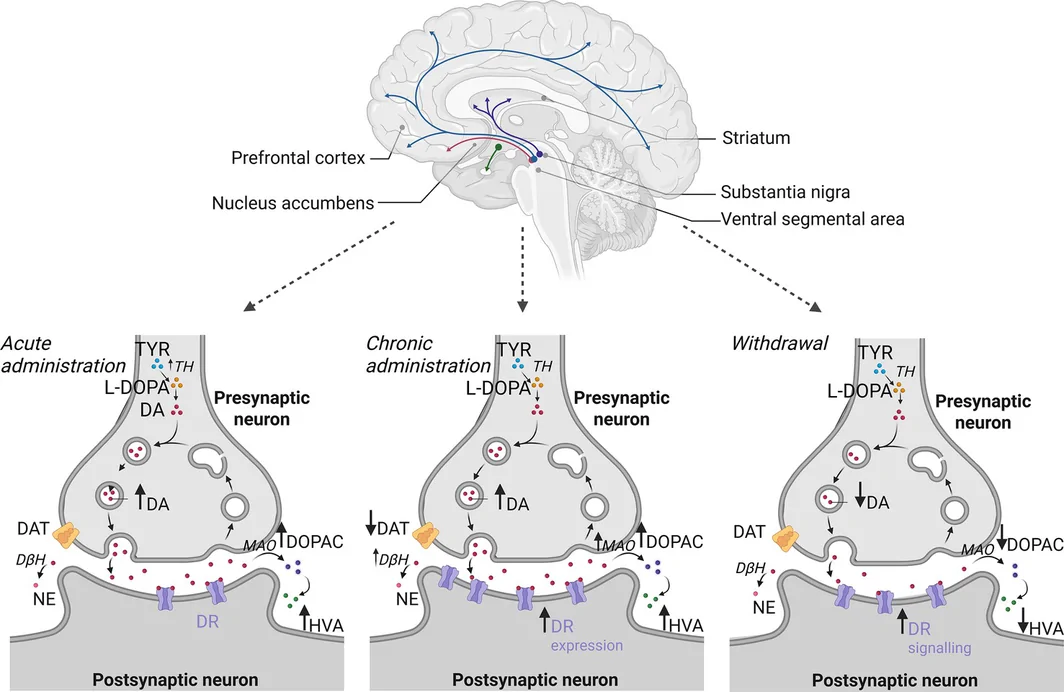
Effects of opioids on the dopaminergic system. Schematic representation of the main dopaminergic pathways in the brain (upper panel), with the mesolimbic pathway shown in pink, the nigrostriatal pathway in purple, the tuberoinfundibular pathway in green, and the mesocortical pathway in blue. The lower panel summarizes the effects of acute and chronic administration of μ‐opioid receptor agonists and opioid withdrawal on the dopaminergic system. Abbreviations: DA, dopamine; DAT, dopamine transporter; DβH, dopamine β‐hydroxylase; DOPAC, 3,4‐dihydroxyphenylacetic acid; DR, dopamine receptor; HVA, homovanillic acid. Arrows indicate increases (↑) or decreases (↓).

### 4.2 Opioids and the Glutamatergic System

#### 4.2.1 Effects of Acute Administration

Opioids significantly modulate the glutamatergic system, which involves glutamate—the brain's primary excitatory neurotransmitter—and plays a central role in addiction‐related processes (Peters and De Vries 2012). Acute activation of MOR or KOR can reduce extracellular glutamate levels in glutamate‐rich regions such as the HIP, Ctx, and NAc, indicating the presence of an opioid–glutamatergic regulatory pathway (Guo et al. 2005; Zhu et al. 1998). At the same time, opioids can activate NMDA receptors, lowering the nociceptive threshold and diminishing opioid analgesic efficacy (Pourzitaki et al. 2018).

The glutamatergic and dopaminergic systems are tightly interconnected. Acute morphine preferentially modulates glutamatergic transmission in cortical‐projecting dopaminergic neurons via AMPA receptors, thereby influencing drug‐seeking and motivational behaviors (Floresco and Magyar 2006; Lane et al. 2008). A single dose of morphine has also been shown to increase mRNA expression of NMDA receptor subunits (NR1, NR2A, NR2B) within hours of administration (Le Grevès et al. 1998). In addition to regulating glutamate release, opioids—including MOR, KOR, and DOR agonists—modulate Ca^2+^ influx through glutamatergic receptor signaling following K^+^‐induced depolarization, thereby influencing neurotransmitter release dynamics (Frank 1998; Vlaskovska et al. 1997). DOR agonists, such as SNC80, suppress activity in hippocampal glutamatergic neurons as well as in brainstem serotonergic and dopaminergic neurons. This effect is likely mediated by increased serotonin levels and activation of 5‐HT₁A receptors, contributing to anxiolytic effects (Grinchii et al. 2023; Rawls and Cowan 2006).

Acute opioid exposure also affects glutamate metabolism. For example, tramadol increases the activity of GDH and GS while decreasing glutaminase (GLS) activity across multiple brain regions, including the Ctx, hypothalamus, HIP, and CB (Chetan et al. 2015).

#### 4.2.2 Effects of Chronic Administration

Chronic opioid exposure generally reduces glutamate levels (Verdejo‐García et al. 2013; Yücel et al. 2007), both directly through presynaptic MOR activation on glutamatergic neurons (Ostermeier et al. 2000; Vogt et al. 2001) and indirectly via dopaminergic modulation of VTA glutamatergic neurons (McGovern et al. 2023; Rada, Mark, et al. 1991; Yamamoto and Davy 1992). However, repeated cycles of opioid use and withdrawal can produce sensitization, characterized by progressively enhanced drug responses. In contrast to acute exposure, chronic MOR activation may increase glutamate release in the LC due to enhanced Ca^2+^ influx during neuronal depolarization (Farahmandfar et al. 2011; Tai et al. 2006; Zhu et al. 1998). This is accompanied by upregulation of cAMP/PKA and PKC signaling pathways, contributing to opioid tolerance (Bie and Pan 2005).

Opioids modulate both presynaptic glutamate release and postsynaptic signaling through AMPA and NMDA receptors in the NAc and VTA. NMDA receptor activation negatively regulates opioid analgesia and contributes to tolerance via receptor upregulation (Chartoff and Connery 2014; Mao 2002; Rodríguez‐Muñoz et al. 2012). Some opioids, such as methadone, may act as NMDA receptor antagonists, thereby enhancing analgesic efficacy (Weschules et al. 2008). Importantly, excessive glutamatergic signaling may lead to excitotoxicity, whereas reduced mGluR2 expression increases sensitivity to opioid reward and exacerbates withdrawal symptoms (Gao et al. 2018; Koob and Volkow 2016).

Chronic opioid use also disrupts the glutamate/GABA–glutamine metabolic axis. Prolonged tramadol exposure increases GDH and GS activity while reducing GLS activity across most brain regions (Caspani et al. 2022; Chetan et al. 2015). Subsequent increases in extracellular glutamate levels may result from downregulation of glutamate transporters such as GLT‐1 and EAAT1 (Farahmandfar et al. 2011; Ozawa et al. 2001; Tai et al. 2006).

#### 4.2.3 Effects of Opioid Withdrawal

Opioid withdrawal markedly enhances glutamatergic activity. Naloxone‐precipitated withdrawal increases extracellular glutamate levels in the NAc and LC, contributing to the development of physical dependence (Aghajanian et al. 1994; Sepulveda et al. 1998; Tokuyama et al. 1998). In parallel, glutamate uptake is increased through upregulation of GLT‐1, particularly in the HIP, likely representing a compensatory response aimed at limiting excitotoxicity (Wang et al. 1998; Xu, Bao, et al. 2003). Increased dynorphin expression during withdrawal activates KORs and further enhances GLT‐1 expression via p38 MAPK signaling, thereby contributing to depressive‐like states (Zan et al. 2021).

Withdrawal also induces pronounced alterations in glutamate receptor expression, including increased levels of AMPA, NMDA, and mGluR subunits. Pharmacological blockade of these receptors has been shown to alleviate withdrawal symptoms (Guo et al. 2009; Salisbury et al. 2021). In the case of MOR agonists such as oxycodone, withdrawal is associated with increased mRNA expression of AMPA receptor subunits GluA1–3, NMDA receptor subunits GluN2A–C, and mGluR subtypes 3, 5, 6, and 8. The involvement of glutamate receptor signaling in withdrawal is particularly pronounced, as antagonists of NMDA receptors and mGluRs can attenuate withdrawal‐related behavioral and physiological symptoms (Guo et al. 2009; Salisbury et al. 2021).

Opioid withdrawal syndrome is also associated with disruptions in neurotransmitter metabolism, including decreased expression of glutamine synthetase and GLS. Notably, these alterations can persist for up to 3 months after morphine withdrawal in rat models, highlighting the long‐lasting impact of opioid exposure on glutamatergic homeostasis (Chen et al. 2007; Ujcikova et al. 2021).

The effects of acute and chronic opioid administration, as well as withdrawal, on the glutamatergic system are summarized in Table 2 and illustrated in Figure 2.

**TABLE 2:** Effects of opioids on the glutamatergic system.

Category	Acute administration	Chronic administration	Withdrawal	Reference
Neurotransmitter levels	↓ GLU levels in the HIP, Ctx, and NAc (MOR and KOR)	↓ GLU levels in the NAc and Ctx (MOR agonists)	↑ GLU in the NAc and LC (MOR agonists)	Aghajanian et al. (1994), Sepulveda et al. (1998), Tokuyama et al. (1998), Zhu et al. (1998), Guo et al. (2005), Yücel et al. (2007), Verdejo‐García et al. (2013), Pourzitaki et al. (2018)
Receptor expression and function	↑ NMDA receptor subunits (NR1, NR2A, NR2B) mRNA expression (MOR agonists)	↑ NMDAR signaling (MOR agonists) ↓ NMDAR activity (methadone)	↑ GluA1–3 (AMPAR), GluN2a‐c (NMDAR), and mGluR 3, 5, 6, 8 expression (MOR agonists)	Le Grevès et al. (1998), Mao (2002), Guo et al. (2009), Rodríguez‐Muñoz et al. (2012), Chartoff and Connery (2014), Salisbury et al. (2021)
Transporters		↓ GLT1 and EAAT 1 function (MOR agonists)	↑ GLT1 expression in the HIP (MOR agonists)	Wang et al. (1998), Ozawa et al. (2001), Xu, Bao, et al. (2003), Tai et al. (2006), Farahmandfar et al. (2011)
Enzymes	↑ GDH and GS activity ↓ GLS activity in the Ctx, HIP, and hypothalamus (MOR agonists)	↑ GDH and GS activity in the Ctx, CER, HIP, and hypothalamus ↓ GLS activity in the CER HIP, and hypothalamus ↑ GLS activity in the Ctx (MOR agonists)	↓ GS and GLS expression in the Ctx (MOR agonists)	Chen et al. (2007), Chetan et al. (2015), Ujcikova et al. (2021), Caspani et al. (2022)

**FIGURE 2:**
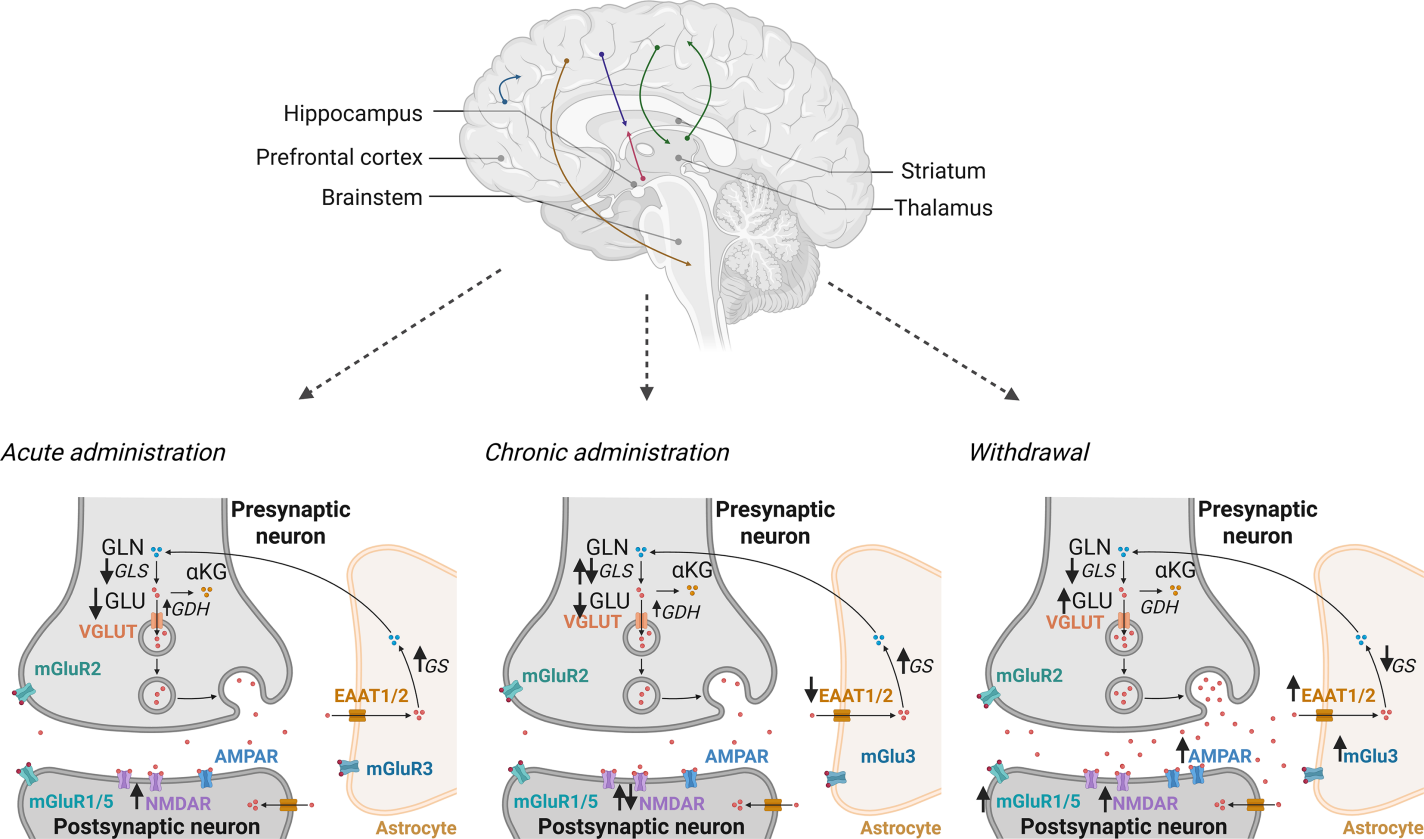
Effects of opioids on the glutamatergic system. Schematic representation of the main glutamatergic pathways in the brain (upper panel), including the corticocortical pathway shown in blue, the corticothalamic pathway in green, the corticostriatal pathway in purple, the corticomesencephalic pathway in brown, and the thalamostriatal pathway in pink. The lower panel summarizes the effects of acute and chronic administration of μ‐opioid receptor agonists and opioid withdrawal on the glutamatergic system. Abbreviations: AMPAR, α‐amino‐3‐hydroxy‐5‐methyl‐4‐isoxazolepropionic acid receptor; αKG, α‐ketoglutarate; EAAT, excitatory amino acid transporter; GDH, glutamate dehydogenase; GLN, glutamine; GLS, glutaminase; GLU, glutamate; GS, glutamine synthetase; mGluR, metabotropic glutamate receptor; NMDAR, N‐methyl‐D‐aspartate receptor; VGLUT, vesicular glutamate transporter. Arrows indicate increases (↑) or decreases (↓). The ↑↓ symbol indicates that the direction of the effect (increase or decrease) depends on the brain region.

### 4.3 Opioids and the GABAergic System

#### 4.3.1 Effects of Acute Administration

Opioids strongly influence the GABAergic system, which plays a central role in regulating dopaminergic activity. Under physiological conditions, GABA exerts inhibitory control over DA release (Kreek et al. 2012). MOR agonists suppress GABAergic interneurons in the VTA, reducing GABA release and thereby disinhibiting dopaminergic neurons, which leads to increased dopamine release in the NAc (Johnson and North 1992; Kelley et al. 1980; Kiyatkin et al. 1993; Xi 2002). At the same time, increased GABA levels have also been reported in the NAc and PFC following opioid exposure, suggesting region‐specific adaptations within the GABAergic system (De Vries and Shippenberg 2002; Schoffelmeer et al. 2001).

MOR‐mediated inhibition of GABA release involves multiple mechanisms, including modulation of potassium conductance and G protein–dependent suppression of vesicular neurotransmitter release (Capogna et al. 1993; Stiller et al. 1996; Vaughan et al. 1997). KOR agonists similarly reduce GABA release, in part by decreasing Ca^2+^ influx at presynaptic terminals (Hjelmstad and Fields 2003).

Acute opioid exposure can also reduce GABA receptor binding, primarily by decreasing high‐affinity receptor sites in the CB, Ctx, and striatum, which may contribute to opioid‐induced antinociception (Rashvand et al. 2014; Ticku and Huffman 1980). In contrast to chronic administration, acute opioid exposure has not been shown to significantly affect GAD activity, suggesting that enzymatic regulation of GABA synthesis is more relevant in long‐term adaptations rather than acute effects (Tzeng and Ho 1978).

#### 4.3.2 Effects of Chronic Administration

Chronic opioid exposure induces significant adaptations in GABAergic neurotransmission, contributing to tolerance, dependence, and addiction (Gao et al. 2007; Hu et al. 2012; Tsuji et al. 1996; Westerink et al. 1996). A key mechanism underlying these changes is the transition from acute inhibition of AC/cAMP signaling to AC superactivation during chronic exposure and withdrawal. This shift leads to increased cAMP production and enhanced GABA release onto dopaminergic neurons in the VTA (Avidor‐Reiss et al. 1996; Ronström et al. 2023).

Altered GABAergic plasticity disrupts dopaminergic signaling and contributes to opioid reward‐related behaviors (Madhavan et al. 2010; Spyraki et al. 1983). Pharmacological activation of GABA‐B receptors can counteract opioid‐induced sensitization and reduce elevated DA levels (Fu et al. 2012; Shippenberg et al. 1996). Chronic opioid exposure also affects GABA metabolism by reducing GAD activity, thereby impairing GABA synthesis and further contributing to neurotransmitter imbalance (Gao et al. 2007; Shi et al. 2020; Szpręgiel et al. 2021; Tzeng and Ho 1978).

#### 4.3.3 Effects of Opioid Withdrawal

Opioid withdrawal is associated with increased GABA release, driven by cAMP‐dependent mechanisms (Bajo et al. 2014; Bonci and Williams 1997; Collier and Francis 1975; Hack et al. 2003). This enhanced GABAergic activity contributes to withdrawal symptoms and can be attenuated by inhibiting cAMP signaling pathways (Madhavan et al. 2010).

Withdrawal is also accompanied by alterations in GABA receptor function. Modulation of GABA‐A receptors influences withdrawal severity, with agonists exacerbating and antagonists reducing symptoms (Capasso and Sorrentino 1997). In contrast, activation of GABA‐B receptors, for example by baclofen, can alleviate withdrawal symptoms (Capasso 1999). In addition, increased GAT‐1 transporter activity enhances GABAergic excitability in the periaqueductal gray (PAG), directly contributing to the expression of withdrawal symptoms (Bagley et al. 2011; Williams et al. 2001).

The effects of acute and chronic opioid administration, as well as withdrawal, on the GABAergic system are summarized in Table 3 and illustrated in Figure 3.

**TABLE 3:** Effects of opioids on the GABAergic system.

Category	Acute administration	Chronic administration	Withdrawal	Reference
Neurotransmitter levels	↓ GABA release in the VTA, PAG, and HIP (MOR agonists)	↑ GABA release in the VTA (MOR agonists)	↑ GABA release in the NAc (MOR agonists)	Collier and Francis (1975), Kelley et al. (1980), Johnson and North (1992), Capogna et al. (1993), Kiyatkin et al. (1993), Avidor‐Reiss et al. (1996), Xi (2002), Hjelmstad and Fields (2003), Kreek et al. (2012), Bajo et al. (2014), Ronström et al. (2023)
Receptor expression and function	↓ GABA receptor binding sites in the CER, Ctx, and striatum (MOR agonists)	↑ GABA receptor binding sites in the pons and medulla ↑ GABA receptor affinity in the CER ↓ in the striatum (MOR agonists)	↑ mRNA expression of ε GABAA subunit in the LC (MOR agonists)	Ticku and Huffman (1980), Sivam et al. (1982), Shippenberg et al. (1996), Capasso and Sorrentino (1997), Capasso (1999), Heikkilä et al. (2001), Xi (2002), Fu et al. (2012)
Transporters			↑ GAT‐1 activity in the PAG (MOR agonists)	Williams et al. (2001), Bagley et al. (2011)
Enzymes		↓ GAD activity (MOR agonists)		Tzeng and Ho (1978), Gao et al. (2007), Shi et al. (2020), Szpręgiel et al. (2021)

**FIGURE 3:**
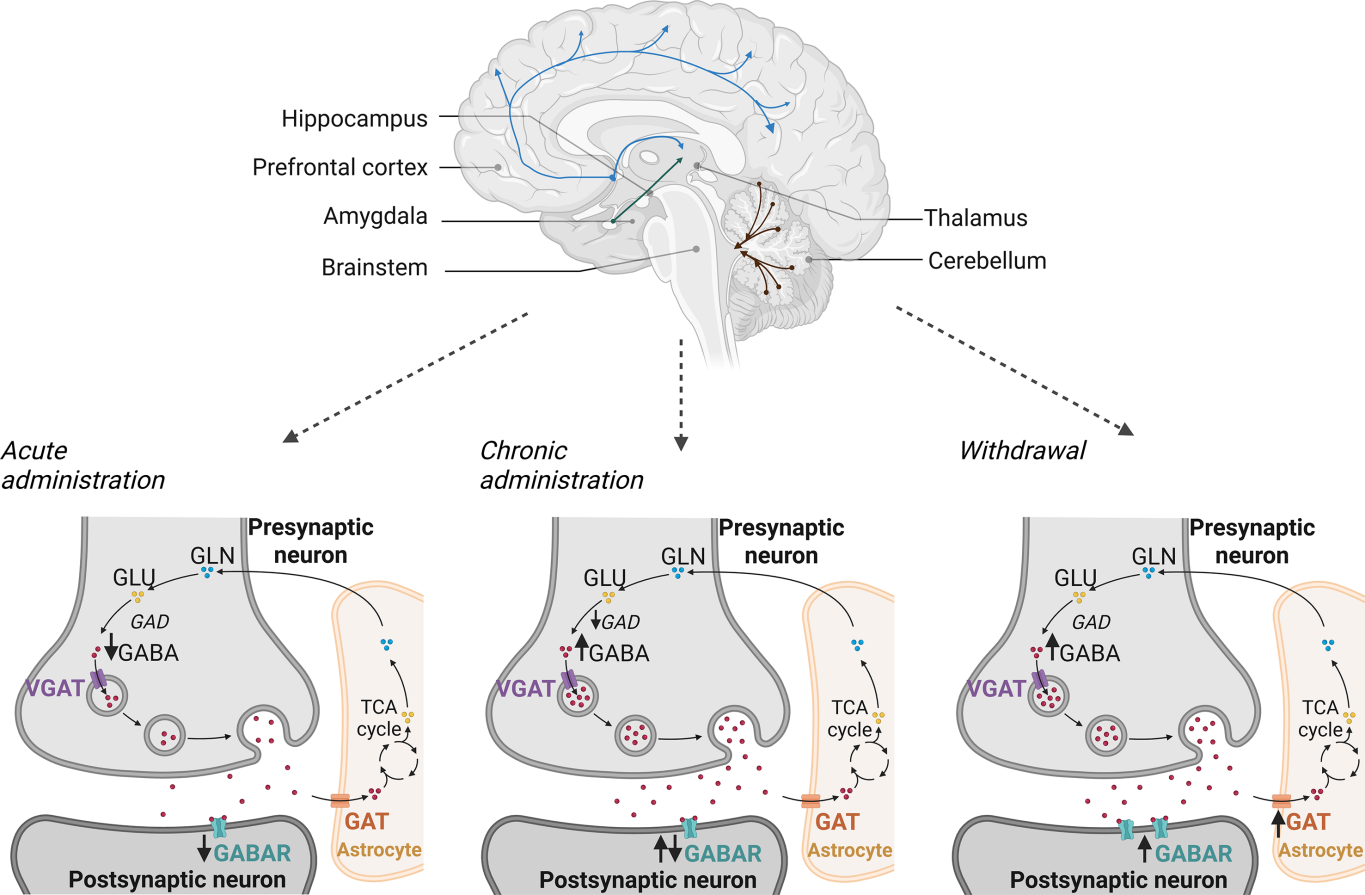
Effects of opioids on the GABAergic system. Schematic representation of the main GABAergic pathways in the brain (upper panel), including thalamic–amygdalar projections shown in green, cortical and hippocampal projections in blue, and the cerebellar pathway in brown. The lower panel summarizes the effects of acute and chronic administration of μ‐opioid receptor agonists and opioid withdrawal on the GABAergic system. Abbreviations: GABA, γ‐aminobutyric acid; GABAR, GABA receptor; GAT, GABA transporter; GLN, glutamine; GLU, glutamate; TCA, tricarboxylic acid; VGAT, vesicular GABA transporter. Arrows indicate increases (↑) or decreases (↓). The ↑↓ symbol indicates that the direction of the effect (increase or decrease) depends on the brain region.

### 4.4 Opioids and the Serotonergic System

#### 4.4.1 Effects of Acute Administration

Opioids also modulate serotonergic neurotransmission. Administration of MOR agonists, such as morphine and heroin, increases serotonin synthesis and release, leading to elevated levels of its metabolite 5‐HIAA, likely through enhanced activity of TPH (Boadle‐Biber et al. 1987; Pérez‐Cruet et al. 1975; Sawa and Oka 1976; Spampinato et al. 1985). This effect may result from reduced inhibitory input secondary to opioid‐induced suppression of GABAergic transmission, a mechanism analogous to that observed in dopaminergic neurons within the VTA (Bloom et al. 1972; Bonci and Williams 1997).

Region‐specific effects have been reported. MOR and DOR agonists increase serotonin release in the dorsal raphe nucleus (DRN) but not in the NAc, whereas KOR agonists reduce serotonin release in both regions. These findings suggest that OR subtypes differentially regulate serotonergic transmission across brain circuits (Tao and Auerbach 2002). Similar to their effects on dopamine signaling, KOR agonists appear to act primarily at presynaptic terminals, modulating neurotransmitter release without significantly altering neuronal excitability (Jolas and Aghajanian 1997; Spanagel et al. 1990; Thompson et al. 2000).

Although acute opioid administration does not appear to directly alter serotonergic receptor expression, functional interactions between these systems are well‐established. Activation of 5‐HT_2_A and 5‐HT_2_C receptors can counteract morphine‐induced increases in extracellular dopamine and reduce dopaminergic neuronal activity (Willins and Meltzer 1998).

#### 4.4.2 Effects of Chronic Administration

Chronic opioid‐induced modulation of the serotonergic system appears to contribute more to antinociception than to reward or dependence mechanisms (Duman et al. 2004; Sasaki et al. 2021; Zhao et al. 2007). Opioid analgesia is partly mediated through serotonergic activation (Nemmani and Mogil 2003). However, prolonged opioid use leads to tolerance and may induce opioid‐induced hyperalgesia, thereby contributing to physical dependence (Mao 2002; Sasaki et al. 2021; Xu, Colpaert, and Wiesenfeld‐Hallin 2003).

Certain MOR agonists, such as tramadol, methadone, and pethidine, increase extracellular serotonin levels by inhibiting the serotonin transporter (SERT), thereby enhancing their analgesic properties. This effect is not observed in structurally distinct opioids such as morphine or oxycodone (Barann et al. 2015; Codd et al. 1995; Rickli et al. 2018; Sotgiu et al. 2009). However, SERT inhibition also increases the risk of serotonin syndrome, particularly with long‐acting agents such as methadone (Baldo and Rose 2020; Fox et al. 2009; Gillman 2005).

Chronic opioid exposure also alters serotonergic receptor function, including facilitation of 5‐HT₁ receptor signaling (associated with anxiolytic and analgesic effects) and inhibition of 5‐HT₃ receptors (linked to nausea, hyperalgesia, and tolerance) (Tao and Auerbach 2002). Additionally, some MOR agonists can interact with 5‐HT_2_A receptors, potentially contributing to withdrawal‐related phenomena (Pang et al. 2016; Rickli et al. 2018).

Serotonin can form covalent bonds with cytosolic and nuclear proteins through a process known as serotonylation. This post‐translational modification can alter protein signaling properties and has been implicated in synaptic plasticity, depression, and neurodegeneration (Bader 2019; Fu and Zhang 2019; Jiang et al. 2021; Muma and K. Kapadia 2020). The impact of opioids on serotonylation has not yet been investigated; however, this mechanism may represent a potentially relevant but currently unexplored pathway in the development of opioid addiction.

#### 4.4.3 Effects of Opioid Withdrawal

Opioid withdrawal produces effects opposite to those observed during drug administration. Reduced serotonergic transmission and decreased TPH expression in the DRN have been reported (Harris and Aston‐Jones 2001; Lunden and Kirby 2013). Enhanced 5‐HT₁A receptor function during chronic exposure diminishes after cessation (Lunden and Kirby 2013; Lutz et al. 2011).

These alterations contribute to mood disturbances during withdrawal, including depressive‐like symptoms that intensify with prolonged abstinence. Dysfunction of the serotonergic system is therefore likely involved in both withdrawal symptomatology and relapse vulnerability (Goeldner et al. 2011; Lunden and Kirby 2013; Renoir et al. 2012).

The effects of acute and chronic opioid administration, as well as withdrawal, on the serotonergic system are summarized in Table 4 and illustrated in Figure 4.

**TABLE 4:** Effects of opioids on the serotonergic system.

Category	Acute administration	Chronic administration	Withdrawal	Reference
Neurotransmitter levels	↑ 5‐HT and 5‐HIAA levels (MOR and DOR agonists) in the DRN ↓ 5‐HT levels in the NAc and DRN (KOR agonists)	↑ 5‐HT and 5‐HIAA levels in the DRN	↓ 5‐HT levels in the DRN	Pérez‐Cruet et al. (1975), Sawa and Oka (1976), Spampinato et al. (1985), Harris, (2021)
Receptor expression and function	Activation of 5‐HT2A and 5‐HT2C receptors counteracts opioid‐induced↑ DA levels in the NAc (MOR agonists)	↑ 5‐HT1R activity, ↓ 5‐HT3R activity (MOR agonists) ↑ binding of MOR agonists to 5‐HT2A receptors	↓ 5‐HT1R activity (MOR agonists)	Willins and Meltzer (1998), Tao and Auerbach (2002), Lutz et al. (2011), Lunden and Kirby (2013), Pang et al. (2016), Rickli et al. (2018)
Transporters		↓ SERT activity (MOR agonists without oxygen bridge structure, e.g., pethidine, tramadol, and methadone)		Codd et al. (1995), Sotgiu et al. (2009), Barann et al. (2015), Rickli et al. (2018)
Enzymes	↑ TPH activity (MOR agonists)		↓ TPH mRNA expression (MOR agonists)	Boadle‐Biber et al. (1987), Lunden and Kirby (2013)

**FIGURE 4:**
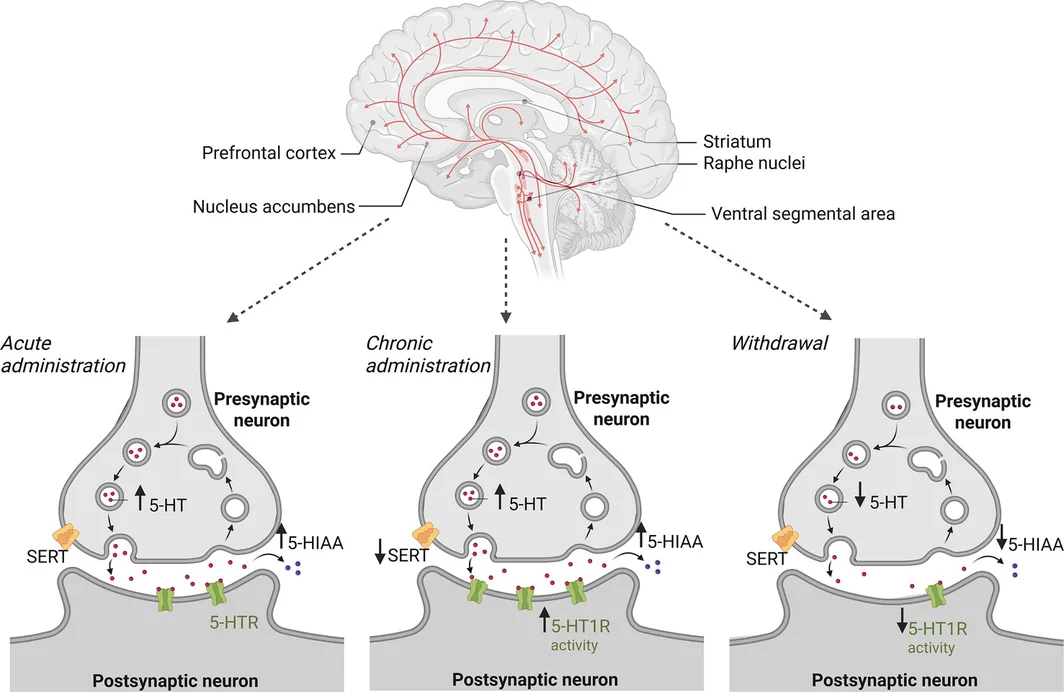
Effects of opioids on the serotonergic system. Schematic representation of the main ascending and descending serotonergic projections (shown in red), originating primarily from the raphe nuclei in the brain (upper panel), and the effects of acute and chronic administration of μ‐opioid receptor agonists and opioid withdrawal on the serotonergic system (lower panel). Abbreviations: 5‐HIAA, 5‐hydroxyindoleacetic acid; 5‐HT, serotonin; 5‐HTR, serotonin receptor; SERT, serotonin transporter. Arrows indicate increases (↑) or decreases (↓).

### 4.5 Opioids and the Cholinergic System

#### 4.5.1 Effects of Acute Administration

The effects of acute opioid exposure on the cholinergic system vary depending on brain region and dose. MOR and DOR agonists inhibit striatal cholinergic interneurons, whereas KOR agonists show minimal effects on these neurons (Arenas et al. 1990). Morphine also suppresses glutamate‐stimulated ACh release in the Ctx, thereby impairing cognitive and arousal‐related processes (Osman et al. 2005). Despite these inhibitory actions, morphine has been reported to increase ACh levels in regions such as the striatum and trigeminal nuclei, likely due to reduced release followed by intracellular accumulation and compensatory dynamics (Green et al. 1976; Taguchi et al. 1993; Zhu et al. 2008). These region‐specific effects may involve interactions with other neurotransmitter systems, particularly GABAergic disinhibition (Johnson and North 1992; Zhu et al. 2008).

Opioids also influence AChE activity. Acute morphine reduces AChE activity in the medulla, whereas tramadol inhibits AChE across multiple brain regions, leading to increased ACh availability (Pandanaboina et al. 2012; Wahba et al. 1987).

Although opioids do not directly act on cholinergic receptors, functional interactions between these systems are well‐established. For example, MOR and DOR agonists suppress dopamine release indirectly by inhibiting cholinergic interneurons, an effect that can mimic nicotinic receptor desensitization. In contrast, KOR‐mediated effects appear largely independent of nicotinic receptor function (Britt and McGehee 2008; Das et al. 1986).

#### 4.5.2 Effects of Chronic Administration

Chronic opioid exposure induces significant adaptations in cholinergic signaling, influencing analgesia, reward, and dependence (Jensen et al. 2018). Changes in ACh release are strongly region‐dependent. Chronic morphine increases ACh release in the HIP and laterodorsal tegmental nucleus, thereby enhancing dopaminergic transmission, while simultaneously decreasing cortical ACh release, a change associated with withdrawal‐like states (Bouaziz et al. 1996; Jhamandas et al. 1975; Jhamandas and Sutak 1974).

Chronic opioid use also alters AChE activity. Increased AChE activity has been observed in the Ctx and medulla, which may contribute to tolerance development. In contrast, reduced AChE activity has been reported in the midbrain and medial habenula, suggesting region‐specific and functionally divergent adaptations within the cholinergic system (Giacobini 2004; Hikida et al. 2003; Neugebauer et al. 2013; Slatkin and Rhiner 2004).

Modulation of KOR signaling further influences cholinergic‐dependent cognitive processes by regulating hippocampal ACh release, thereby affecting learning and memory functions (Hiramatsu et al. 1998; Serra et al. 1997; Takahashi et al. 2018).

Muscarinic receptors, particularly the M1 subtype, also interact functionally with opioid systems. Blocking or genetically deleting M1 receptors enhances morphine‐induced analgesia and reduces conditioned place preference, indicating that cholinergic signaling contributes to both pain modulation and reward‐related learning processes (Carrigan and Dykstra 2007; McKendrick and Graziane 2020).

#### 4.5.3 Effects of Opioid Withdrawal

Opioid withdrawal is associated with increased cholinergic activity, particularly elevated ACh levels in the NAc, contributing to withdrawal symptoms (Rada, Pothos, et al. 1991; Rada et al. 1996). This may reflect accumulated ACh during chronic exposure followed by excessive release upon withdrawal. Pharmacological modulation of cholinergic signaling can influence withdrawal severity. Clonidine reduces ACh release and alleviates withdrawal symptoms (Bhargava and Way 1975; Pinsky et al. 1973).

Opioid withdrawal is associated with increased cholinergic activity, particularly elevated ACh levels in the NAc, which contribute to withdrawal symptomatology (Rada, Pothos, et al. 1991; Rada et al. 1996). This increase may reflect intracellular accumulation of ACh during chronic opioid exposure, followed by excessive release upon drug cessation. Pharmacological modulation of cholinergic signaling can significantly influence the severity of withdrawal. For example, clonidine reduces ACh release and alleviates withdrawal symptoms, likely through its inhibitory effects on central noradrenergic and downstream cholinergic activity (Bhargava and Way 1975; Pinsky et al. 1973).

Muscarinic receptor signaling also plays an important modulatory role. Deletion of M5 muscarinic receptors reduces both opioid reinforcement and withdrawal symptoms, suggesting a role in reward–withdrawal circuitry. Conversely, muscarinic agonists such as arecoline may attenuate withdrawal‐related disturbances, further supporting the involvement of muscarinic receptor pathways in opioid dependence and withdrawal regulation (Basile et al. 2002; Buccafusco et al. 2000; Neugebauer et al. 2013).

The effects of acute and chronic opioid administration, as well as withdrawal, on the cholinergic system are summarized in Table 5 and illustrated in Figure 5.

**TABLE 5:** Effects of opioids on the cholinergic system.

Category	Acute administration	Chronic administration	Withdrawal	Reference
Neurotransmitter levels	↓ ACh release in the Ctx and striatum ↑ ACh levels in the striatum and trigeminal nuclei (MOR agonists)	↑ ACh release in the HIP and LDTg enhancing DA transmission in the VTA (MOR agonists) ↓ ACh release in the Ctx and striatum (MOR, KOR, and DOR agonists)	↓ ACh release in the Ctx and striatum ↑ ACh levels in the NAc (MOR agonists)	Jhamandas and Sutak (1974), Jhamandas et al. (1975), Green et al. (1976), Mulder et al. (1984), Arenas et al. (1990), Rada et al., (1991), Bouaziz et al. (1996), Imperato et al. (1996), Rada et al. (1996), Schoffelmeer et al., 1997, Osman et al. 2005, Zhu et al. 2008
Receptor expression and function	No data available	No data available	No data available	
Transporters	No data available	No data available	No data available	
Enzymes	↓ AChE activity in the medulla oblongata, Ctx, and HIP (MOR agonists)	↑ AChE activity in the medulla oblongata and Ctx ↓ AChE activity in the medulla oblongata and medial habenula (MOR agonists)	↑ AChE activity in the medial habenula (MOR agonists)	Wahba et al. (1987), Pandanaboina et al. (2012), Neugebauer et al. (2013), Elwy and Tabl (2017)

**FIGURE 5:**
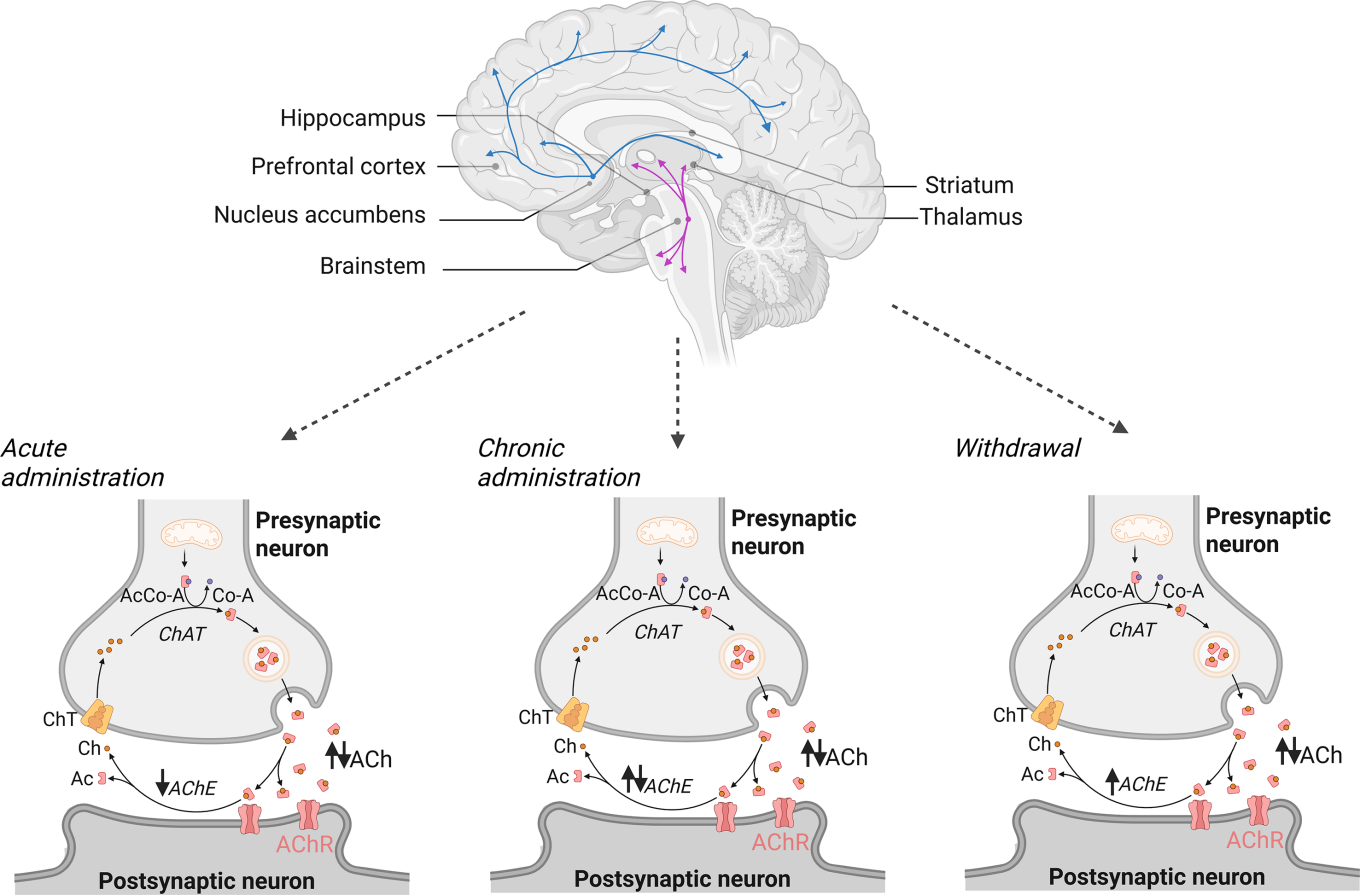
Effects of opioids on the cholinergic system. Schematic representation of the main cholinergic pathways in the brain (upper panel), including basal forebrain projections shown in blue and brainstem projections in pink, and the effects of acute and chronic administration of μ‐opioid receptor agonists and opioid withdrawal on the cholinergic system (lower panel). Abbreviations: Ac, acetate; AcCoA, acetyl‐coenzyme A; ACh, acetylcholine; AChE, acetylcholinesterase; AChR, acetylcholine receptor; Ch, choline; ChT, choline transporter; ChAT, choline acetyltransferase; CoA, coenzyme A. Arrows indicate increases (↑) or decreases (↓). The ↑↓ symbol indicates that the direction of the effect (increase or decrease) depends on the brain region.

## 5 Conclusions, Translational Challenges, and Perpectives

Opioids exert widespread and complex effects across multiple neurotransmitter systems, including dopaminergic, glutamatergic, GABAergic, serotonergic, and cholinergic pathways. These effects vary across acute exposure, chronic use, and withdrawal and involve changes in receptor expression, intracellular signaling, synaptic plasticity, and circuit function. Acute opioid administration enhances dopaminergic signaling through direct receptor‐mediated effects and disinhibition of dopaminergic neurons, thereby reinforcing reward pathways. Chronic exposure induces persistent neuroadaptations that contribute to tolerance, dependence, and addiction, whereas withdrawal is characterized by reduced dopaminergic activity and compensatory hypersensitivity, contributing to negative affective states and relapse vulnerability.

Opioids also substantially alter glutamatergic, GABAergic, serotonergic, and cholinergic neurotransmission. Chronic exposure and withdrawal disrupt glutamatergic plasticity and excitatory signaling, contributing to tolerance, sensitization, maladaptive drug‐related learning, and withdrawal‐associated negative affect. GABAergic adaptations, including changes in cAMP‐dependent signaling, contribute to neuronal hyperexcitability and withdrawal symptoms. Serotonergic signaling contributes to analgesia and mood regulation but is also altered during chronic exposure and withdrawal, potentially exacerbating affective symptoms. Cholinergic neurotransmission is modulated in a region‐specific manner and may influence cognition, reward processing, and withdrawal through changes in ACh release, AChE activity, and muscarinic receptor function. Collectively, these findings highlight the extensive interdependence of opioid effects across neurotransmitter systems rather than isolated dysfunction within individual pathways.

This complexity is particularly relevant to the reward circuitry, where dopaminergic, glutamatergic, GABAergic, serotonergic, and cholinergic networks interact with stress‐ and memory‐related circuits. These interconnected systems therefore represent potential targets for understanding the neurobiological mechanisms underlying OUD and for developing novel therapeutic strategies. However, their substantial overlap and compensatory adaptations create important translational challenges, and the therapeutic value of targeting individual neurotransmitter systems remains limited compared with established treatments.

Dopaminergic signaling has received considerable attention because chronic opioid exposure produces marked changes in dopamine function. During withdrawal, reduced dopaminergic activity contributes to anhedonia, diminished motivation, and negative affect, whereas opioid‐associated cues can evoke strong dopaminergic responses that promote drug‐seeking and relapse. Dopamine D2‐ and D3‐receptor systems, particularly D3 receptors, have been investigated as potential targets for reducing reward‐related effects and cue‐induced craving (de Guglielmo et al. 2017; Nickols et al. 2023). Nevertheless, dopamine‐targeting medications have not demonstrated sufficient clinical efficacy to become evidence‐based first‐line treatments for OUD.

Glutamatergic signaling is similarly altered by chronic opioid exposure and withdrawal. Increased glutamatergic activity, including within the NAc, may contribute to aversive states and maladaptive drug‐related learning. Consequently, glutamate‐modulating strategies, including NMDA receptor antagonism, memantine, and topiramate, have been investigated as approaches to reduce withdrawal symptoms or relapse (Comer and Sullivan 2007; Jain et al. 2011; Pysiewicz et al. 2025). However, clinical evidence for these interventions remains considerably weaker than that supporting established pharmacological treatments for OUD.

As noted above, GABAergic adaptations also contribute to the neurobiological consequences of chronic opioid exposure and withdrawal. GABAergic agents such as benzodiazepines, zolpidem, and related compounds have been investigated for the management of withdrawal‐associated anxiety, pain, and sleep disturbances (Bueschbell et al. 2021; Martin et al. 2023). However, it should be noted that these substances have abuse potential, which limits their clinical utility, and they have not replaced established pharmacological treatments for OUD. Moreover, concomitant use of GABAergic or other CNS depressant medications with opioids requires caution because of the potential for additive respiratory depression.

Serotonergic and cholinergic systems represent additional, but less well‐established, therapeutic targets. Serotonergic dysfunction may contribute to depression, anxiety, and other psychiatric symptoms associated with opioid dependence, while serotonergic agents have also been investigated for the management of specific withdrawal symptoms (Farren and O'Malley 2002; Chu et al. 2017). However, manipulation of serotonin signaling alone has not demonstrated efficacy comparable to established OUD treatments. Potential pharmacological interactions should also be considered, including the uncommon risk of serotonin toxicity when serotonergic medications are combined with methadone or buprenorphine. Cholinergic mechanisms may influence withdrawal, reward‐related learning, and relapse through their effects on ACh release and dopaminergic signaling, but pharmacological modulation of this system remains largely experimental (Jensen et al. 2018).

Overall, although dopaminergic, glutamatergic, GABAergic, serotonergic, and cholinergic systems provide important insights into the neurobiology of OUD and represent potential therapeutic targets, the evidence supporting their pharmacological manipulation remains substantially weaker than that for established treatments such as methadone and buprenorphine. These systems should therefore be considered primarily as complementary or experimental targets rather than replacements for evidence‐based first‐line therapies. A major translational challenge is to determine whether selective modulation of specific receptor subtypes, intracellular signaling pathways, or circuit‐level interactions can provide therapeutic benefits without disrupting physiological neurotransmission or introducing additional adverse effects. Future research should therefore move beyond the study of individual neurotransmitter systems toward an integrated, circuit‐level understanding of opioid‐induced neuroadaptations. Greater attention to the dynamic interactions among reward, stress, and memory circuits may facilitate the development of multimodal, mechanism‐based interventions capable of addressing both the positive and negative motivational states that contribute to opioid dependence and relapse. Such approaches may ultimately lead to more precise and effective strategies for preventing relapse and improving clinical outcomes in OUD.

## Author Contributions

**Daniela Novakova:** writing – original draft, conceptualization. **Eliska Pritulova:** writing – review and editing, funding acquisition. **Jiri Novotny:** writing – review and editing, project administration, conceptualization, resources, supervision.

## Funding

This work was supported by Charles University GA UK project (no. 148524) and Charles University Institutional Research Fund (SVV‐260790).

## Conflicts of Interest

The authors declare no conflicts of interest.

## Data Availability

Data sharing is not applicable to this article as no datasets were generated or analyzed during the current study.
